# Patterned Nanostructures on Cathodes: A Pathway to Stronger, High-Energy, High-Power Li-Ion Batteries

**DOI:** 10.1021/acsnano.5c07146

**Published:** 2025-11-03

**Authors:** Mohammad Zakertabrizi, Farshad Bozorgmehrian, Myunghwan Jeong, Ehsan Hosseini, Victor Ponce, Hamed Fallahi, Saeed Bahadorikhalili, Hadi Nasrabadi, Dorrin Jarrahbashi, Homero Castaneda, Amir Asadi

**Affiliations:** † Department of Mechanical Engineering, 14736 Texas A&M University, College Station, Texas 77843, United States; ‡ Department of Engineering Technology and Industrial Distribution, Texas A&M University, College Station, Texas 77843-3367, United States; § Department of Materials Science and Engineering, Texas A&M University, College Station, Texas 77843-3367, United States; ∥ Department of Petroleum Engineering, Texas A&M University, College Station, Texas 77843-3367, United States

**Keywords:** functional multimaterials, self-assembly, spray coating, cathode patterning, interfacial morphology

## Abstract

Conventional cathodes are produced by layering materials on flat, two-dimensional substrates, imposing a trade-off between capacity retention, energy and power output, and mechanical stability. Herein, we employ programmable spray-deposition manufacturing to introduce flexibility through enabling form factors and tailored morphological nanostructures to overcome this limitation. This tailorability is achieved by creating distinct patterns—disk and ring formations—on the electrode using spray-deposition. The pattern formation is controlled by the self-assembly of lithium iron phosphate (LFP) and reduced graphene oxide (rGO), mixed at tailored mass ratios, and deposited using an in-house spray system onto an aluminum current collector. We show that when active materials are assembled into repeatable, spatially controlled patterned architecture, the resulting structure amplifies certain functionalities such as capacity retention, energy, and power density or interfacial adhesion and structural cohesion. Specifically, disk-patterned cathodes exhibit lower charge transfer resistance, faster kinetics, higher energy and power density, and cyclic stability, whereas ring-patterned cathodes provide superior interfacial adhesion and cohesion compared to disk-patterned cathodes. Interestingly, combining both patterns yields a cathode that simultaneously exhibits superior capacity, cyclic stability, and interfacial adhesion compared with either the disk- or ring-patterned configurations alone. We use molecular dynamics to show that the disk pattern’s superior ion mobility and diffusion kinetics come with a higher chance of trapping lithium ions, whereas the less dense ring pattern, though lower in capacity, can preserve it over longer cycles. Strategically integrating these patterns enables a synergistic multifaceted cathode design to overcome classical engineering trade-offs between electrochemical functionality and mechanical stability.

## Graphical abstract

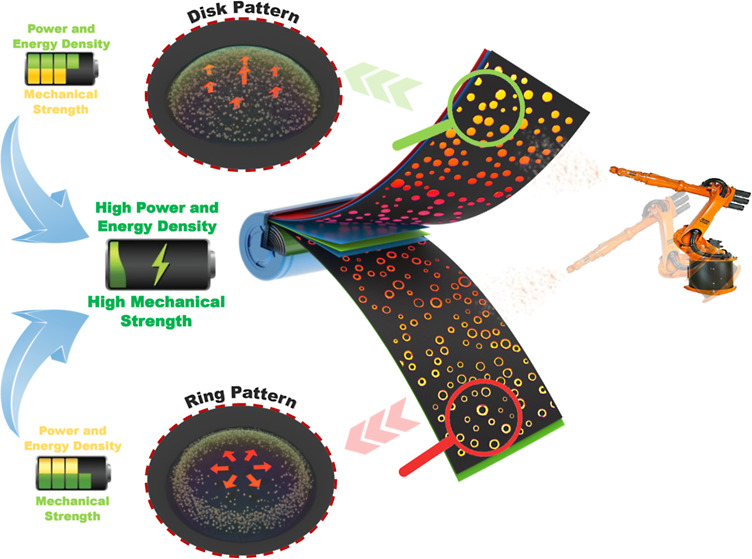

The modern world is shaped by its reliance on electrical power and portability driven by rapid advancements in battery capacity and efficiency. Remarkably, the energy density of lithium-ion batteries has seen a 4-fold increase from 1992 to 2005, a testament to the relentless pursuit of improvement within this field.^1^ With a theoretical capacity of 170 mAh g^–1^, impressive longevity, and consistent operating voltage, lithium iron phosphate (LFP) cathodes have led to the electrification of vehicular transport.^2,3^ Still, they are limited by their low electrical conductivity (10^–9^ S cm^–1^) and intrinsic slow lithium-ion diffusion (10^–14^ cm^2^ s^–1^),^3,4^ partly due to the traditional slurry-based casting technique that prioritizes easy processing and upscaling. The resulting layered structure inherently ties power and stored energy in an inverse relationship: higher stored energy and capacity necessitates adding more active material and increasing the thickness of the structure, which consequently extends the diffusion distance and reduces the power output. This is addressed by reducing the size of LFP particles, which shortens diffusion pathways within the electrode–electrolyte interface—increasing the power density—partially offset by a reduction in energy density due to reduced compaction and accelerated side reactions.^5^ As a countering measure, a conductive coating is applied over the LFP particles. This coating forms an ion/electron-conductive network, while simultaneously inhibits particle aggregation^6^ and prevent side reactions.^7^ Conductivity within the three-dimensional space between the active particles is further enhanced by additives, usually in the form of carbon nanomaterials, whose benefits are weighed against the additional weight and volume they introduce to the cathode.^8^

The mixture of coated LFP and additives can be applied to the target surface through spray coating—an alternative to slurry casting—that offers greater design flexibility: instead of casting a single-layer, spray coating progressively deposits multiple layers, potentially with different compositions, facilitating the fabrication of more intricate electrode designs. While spray coating as a potent delivery system has been previously explored,^9^ its integration with patterning—modifications in the third dimension—as an effective tool for shaping the electrochemistry and mechanical stability of the cathode remains unexplored. The main approaches to patterning require postmanufacturing surface treatments or premanufacturing structured templates,^10^ both too expensive and complex to expand in scale. We propose using self-assembly as an adjustable fabrication method, with promising control over the exact shape of the patterns left as postevaporation deposits from an in-house supercritical spray-deposition system.^11,12^ In our previous work,^13^ we showed that the flow and interactions between the nanomaterials and dispersant (amphiphilicity) within each droplet, controlled by the intermolecular forces, can shift the pattern between a coffee ring shape and a disk shape. Stronger interaction with the dispersant (stronger hydrophilicity) leads to better integration with the internal microflow within the droplet and accumulation of particles on the edges. In contrast, weaker integration with the dispersant (stronger hydrophobicity) leads to the formation of larger conglomerates that do not move with the internal microflow, creating a uniform scattered pattern all over the surface area of postevaporated droplets as they settle. The landing surface covered by an array of these patterns manifests significant shifts in interlayer properties and thus macroscopic functionalities^14^ and mechanical properties.^12^

In this work, we investigate manipulating surface morphology to regulate cathode performance: supercritical N_2_ spray-deposition of patterned cathode materials deposits on an aluminum substrate, in which cathodes are composed of all-ring, all-disk, and combined ring–disk patterns. We have shown that employing supercritical atomization for deposition of nanomaterials allows controlling the uniformity and the size of carrier droplets and thus the postevaporated patterns within few micron resolution.^11,13,15^ We report that a controlled transition from disk to ring patterns in spray-deposited cathodes is enabled by adjusting the LFP:rGO (reduced graphene oxide) weight ratio from 3:1 to LFP:rGO 8:1. We tracked the shift in patterned deposit shapes and its correlation with the electrochemical–mechanical performance of the cathodes. Ring-patterned cathodes show higher interfacial adhesion to the substrate and thus further delamination resistance, whereas disk-patterned cathodes yield higher capacity retention and maximum output power and energy density. We demonstrate that while the ring-patterned cathode trails behind the faster kinetics of rGO-paved pathways in the disk-patterned cathode in the electrochemical indices, their denser structure exhibits structural resilience, and the ring form can project higher cohesion in the electrode–substrate/electrolyte interface. Notably, cathodes with combined disk–ring patterns exhibit enhanced mechanical adhesion, higher capacity retention, and improved ion diffusion compared to individual ring and disk patterns, demonstrating a synergistic effect arising from the integration of both morphologies. These findings position ring–disk patterning as a key design factor, leveraging distinct properties to overcome traditional mechanical–functionality trade-offs.

## Results and Discussion

### Morphology of Patterned 3D Nanostructured Cathodes

Deposited 3D nanostructures—ring and disk-shaped patterns with premeditated size, height, and spacing—are postevaporation self-assembled traces of LFP:rGO suspension with a specified weight fraction. The droplets carrying LFP:rGO were deposited either manually (for validation) with an average diameter size of 1600 μm or generated through an in-house supercritical N_2_ spray system (Figure 1a) to fabricate cathodes. We found that changing the composition of the suspension, LFP:rGO going from 3:1 to 8:1, and thus amphiphilicity, uniformly alters the final form of the deposits, transitioning from disk to ring. This follows the conclusion established in our previous work, where we showed that postevaporation form is mainly a result of the balance between particle–particle and dispersant–particle–substrate interactions.^15^ Here, the strong nonbonding LFP–rGO interaction (e.g., van der Waals, electrostatic) creates a multimaterial dispersing system, where the interaction with the dispersant is governed by the amphiphilicity of LFP:rGO controlled by their mass fraction. Lower rGO presence shifts the polarity within the LFP–rGO system toward a stronger interaction between the multimaterial system and the dimethylformamide (DMF) solvent. The stronger interaction with the dispersant means particles are more likely to follow the capillary flow toward the pinned contact line, ultimately forming a ring-shaped deposit (Figure 1b). Increasing the presence of rGO—the particle with less polarity—leads to a weaker interaction with the polar solvent. As a result, the sedimentation and Marangoni flows overcome the capillary flow within the droplet and prevent the dispersed particles from being distributed toward the contact line. As the dispersant evaporates, the LFP–rGO phase deposits in the middle of the droplet, leaving a disk-like formation on the substrate (Figure 1c). The 3D representation of the mapped deposits observed in the SEM images fully corresponds to the ring and disk patterns. The deposits with the ring pattern are observed to be scattered in a pinned line encompassing the base of the droplet (Figure 1d). Increasing the rGO presence transforms the previously observed ring-like pattern into a distinctive disk pattern, where the majority of the dispersed multimaterial phase accumulated over the base of the droplet (Figure 1e).

**Figure 1:**
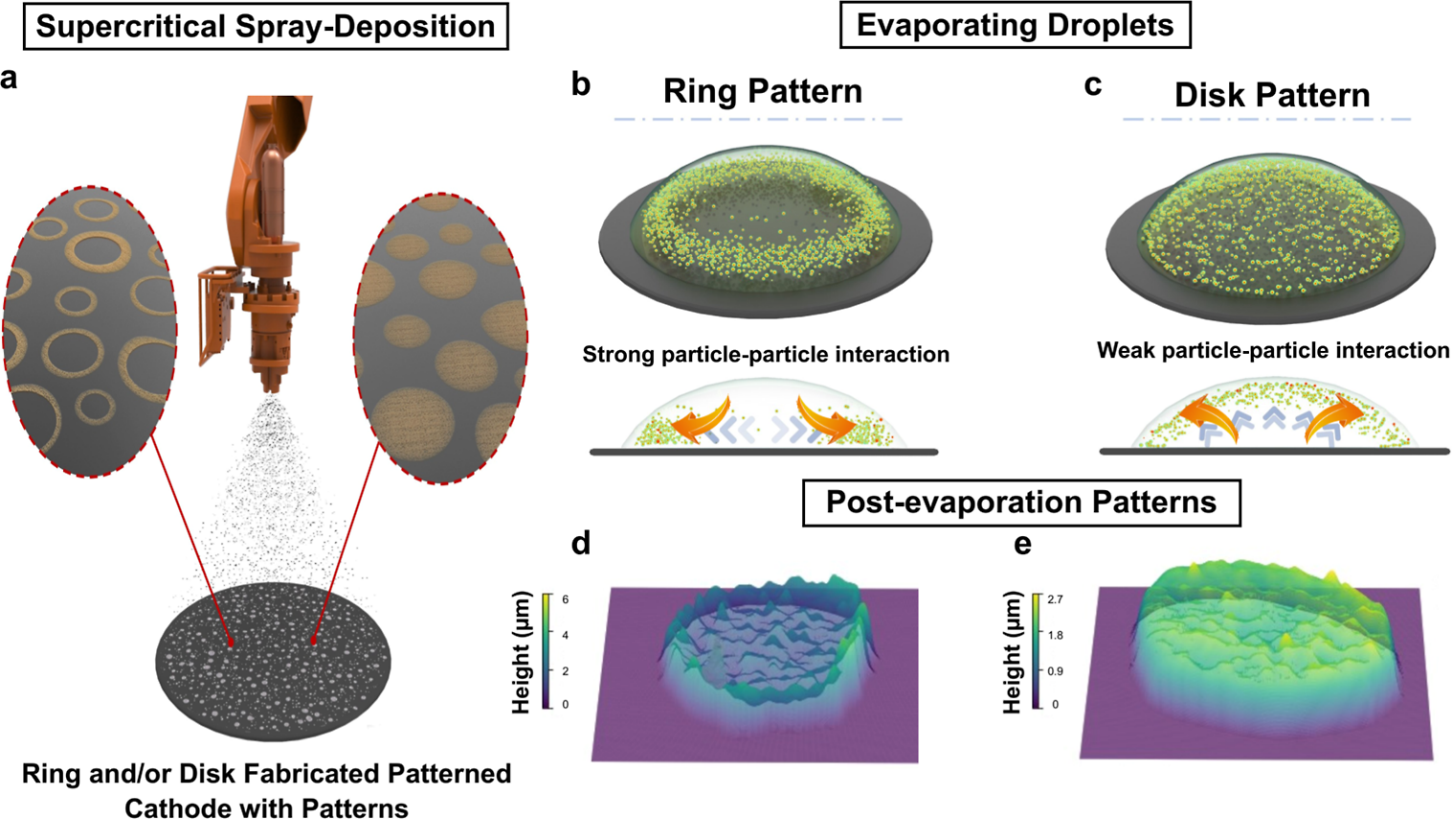
(a) Schematic illustration of the in-house spraying system depositing droplets with different compositions that dry into (b) ring or (c) disk patterns, depending on balance of flows within the droplet. Deposits dry into either (d) ring, where deposits are concentrated along a pinned line, or (e) disk patterns, where the deposits are packed within the perimeter. 3D representations in (d,e) are constructed from SEM images.

SEM images of the postevaporation deposits clearly underline the clear, distinct shift in the distribution of deposited materials in the LFP:rGO 3:1 (disk) and 8:1 (ring) specimens (Figure 2a,b). This disparity in the postevaporation pattern shape is a result of the shift in the interactions within the dispersant, governed by the polarity, size, and surface area of the dispersed particles; for instance, higher rGO presence—the particle with the higher surface area but lower polarity—weakens the affinity between the LFP–rGO and the polar dispersant, which coalescing with increased agglomeration caused by the higher surface area of rGO results in sedimentation overcoming other internal flows within the drying droplet. The result is a disk-shaped pattern in the LFP:rGO 3:1 specimen, where a higher number of particles accumulated toward the center, and bright dots—indicating the presence of LFP—are sparser and smaller. SEM images reveal that when the rGO is reduced in the LFP:rGO 8:1 system, the postevaporation pattern shifts to a ring pattern with a clear distinction between the peripheral and the central area. With reduced rGO presence, the visibility of bright dots increases as fewer LFP particles are obscured by the rGO layer.

**Figure 2:**
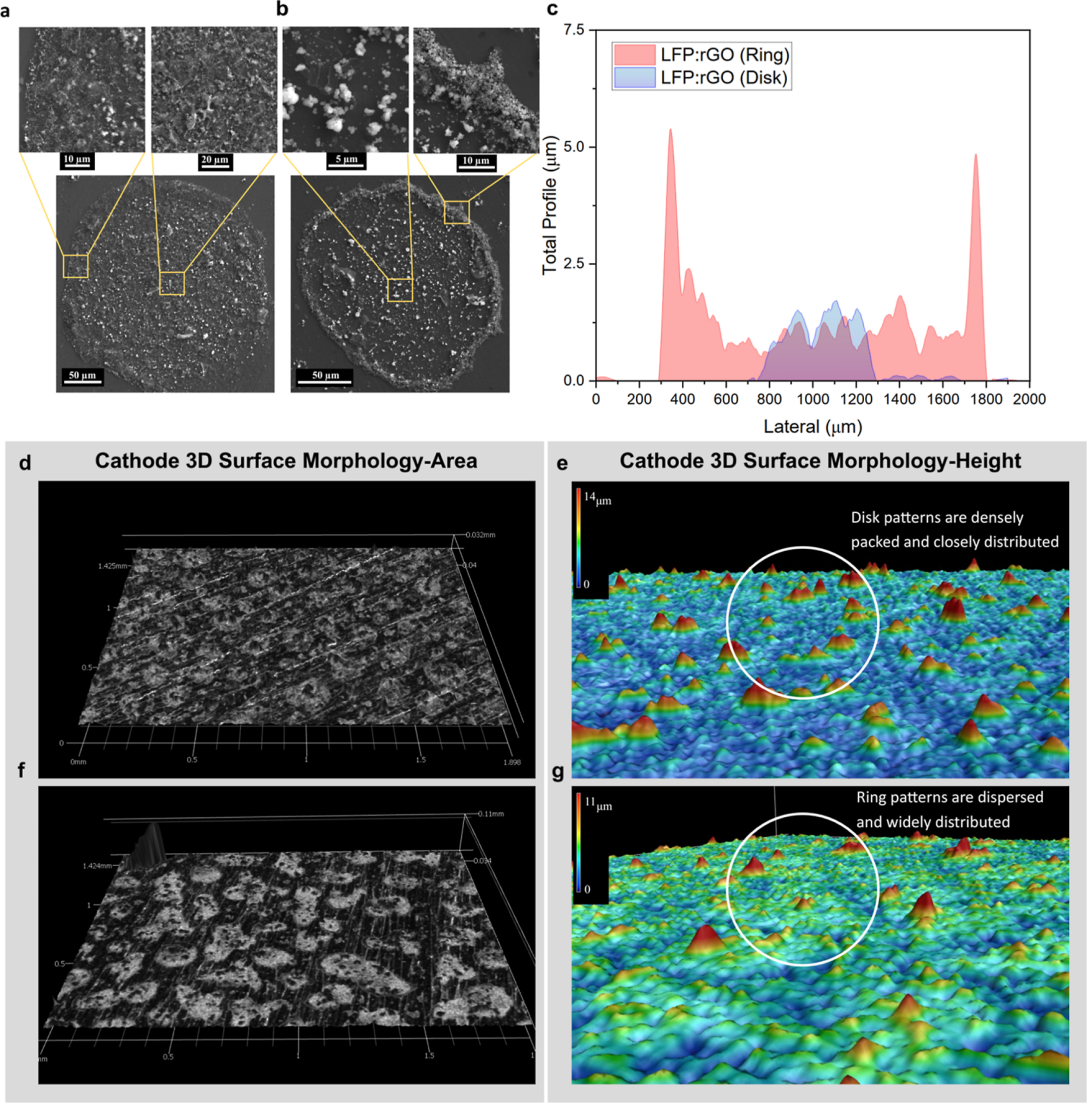
SEM image of (a) disk and (b) ring, where the center and periphery are better shown in inset pictures for both patterns. (c) Height horizontal profile of the postevaporation ring and disk patterns. 3D microscopy images of (d) disk, with the corresponding (e) disk height profile and (f) ring, with the corresponding (g) ring height profile.

We further examined the distribution of deposits along the surface using a profilometer (Figure 2c). The shape of the LFP:rGO 3:1 deposits resembles a nonuniform disk shape, with the height at 1 μm on average, spanning over 500 μm. This shape stands in contrast to the LFP:rGO 8:1 ring pattern, where the particles are mostly deposited on the periphery that extends ∼750 μm from the center. The peripheral line is as high as 5.5 μm, more than twice the average height over the plateau area. A comparison of the two profiles reveals that within a smaller deposit diameter, the disk formations reach heights similar to the inner region of the ring (at ∼1.5 μm). However, the inner region of the ring is enclosed by a surrounding wall that rises more than three times higher than the plateau. While both patterns exhibit a similar morphology on the plateau, the ring-like pattern is characterized by the distinct tall circumferential walls. This suggests that a significant portion of the Li^+^ ions within the ring-patterned cathode might have shorter physical diffusion pathways within the electrode–electrolyte interface. To observe the collective presence of these patterned deposits covering the collector, we employed a 3D microscopy imaging technique to examine the collector after four sprays (Figure 2d–g). The pictures reveal uniformly distributed scattering of the deposits, covering the surface for both the ring and disk formations. When comparing the 3D representation of the images, they showed that the disk patterns are more numerous and densely packed, while the ring patterns are more scattered. Otherwise, we observed that the deposits frequently exhibit a consistent height, indicating uniform deposition of patterns across the collector’s surface. We hypothesize that these patterns can be used to customize the electrode–electrolyte interface, which can affect the microscale kinetics and mechanical stability at the electrode–electrolyte interface. The design decision hinges on the proportion of disk-patterned deposits, which feature higher transport and conductivity, and ring-patterned deposits, which offer greater surface area and structural cohesion.

We used EDS images, which mark the carbon (Figure 3a­(ii),b­(ii)) and phosphorus (Figure 3a­(iii),b­(iii)) elements corresponding to LFP and rGO, respectively, to illustrate the postevaporation concentration distribution of the LFP–rGO system across the deposit surface. In both cases, the overlapping presence of phosphorus and carbon atoms confirms the full integration of the LFP–rGO into a multimaterial system. This further highlights the distinction between ring and disk patterns, as the accumulation of LFP–rGO on the periphery (Figure 3a) transitions toward a more uniform distribution across the entire deposition area (Figure 3b) as the share of rGO in the multimaterial system increases. We used FTIR spectroscopy to characterize the LFP:rGO cathodes (Figure 3c). Both disk and ring exhibit the characteristic vibrational bands of their constituent components; the major markers are around 937 cm^–1^ and 465 cm^–1^ for P–O and Fe–O from the LFP structure. Minor changes in the position and prominence of these bands are due to the change in the rGO content, which has a less pronounced spectral signature. To remedy this, we used Raman spectroscopy to take a closer look at the potential changes in the chemistry of the carbon structure of rGO within the cathode (Figure 3d). The prominent two peaks observed at ∼1370 cm^–1^ and 1619.2 cm^–1^ maintain a near consistent intensity ratio (*I*_D_/*I*_G_) despite a significant decrease in their overall intensity in the ring specimen due to the higher presence of LFP, which has a weaker Raman presence due to its relatively lower polarity. The XRD results support these findings, as the characteristic diffraction peaks of LFP remain prominent in the composite cathodes, indicating minimal changes in crystallinity and consequently no significant chemical alterations (Figure 3e).

**Figure 3:**
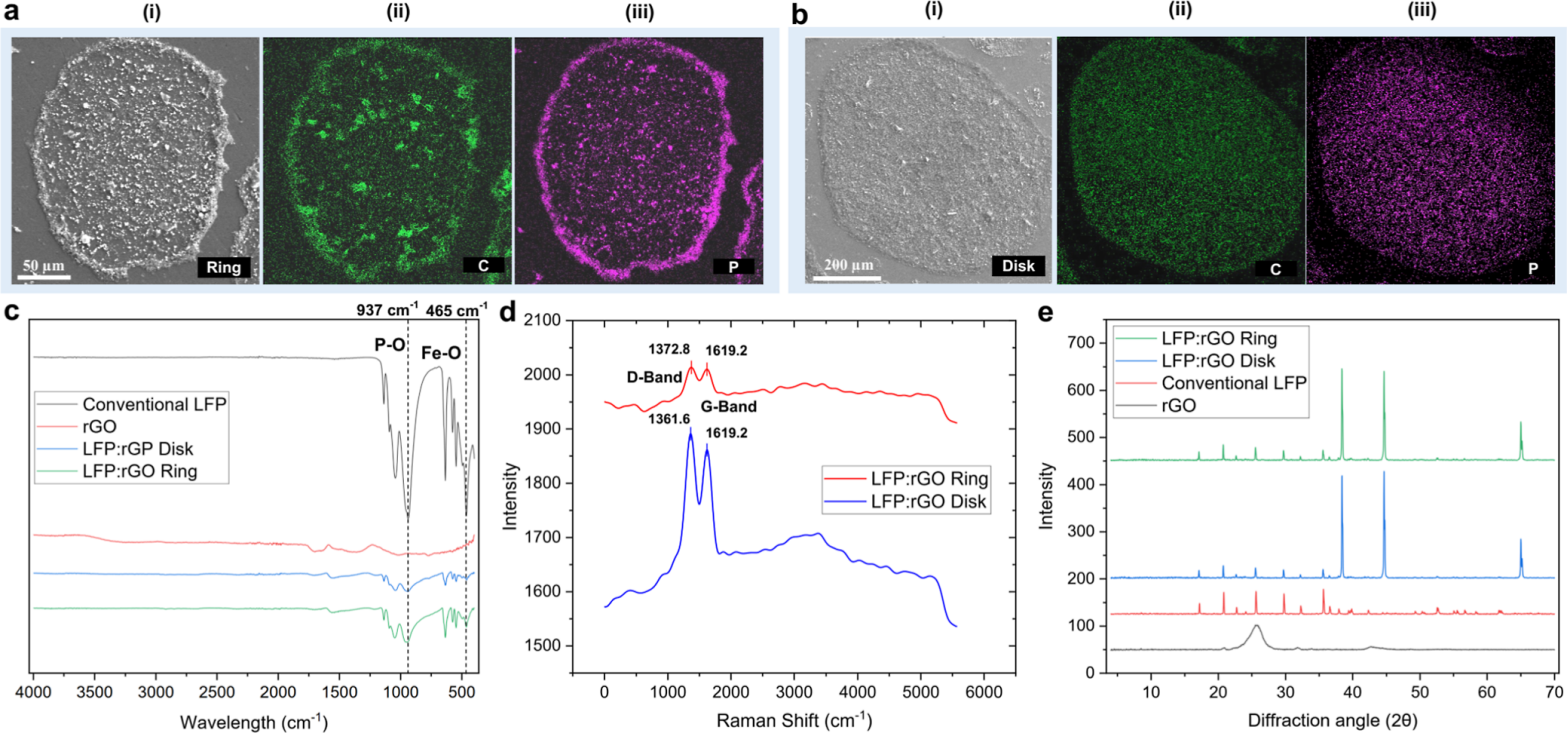
SEM images of (a­(i)) ring- and (b­(i)) disk-patterned deposits. EDS characterization: (a­(ii)) carbon and (a­(iii)) phosphorus for ring and (b­(ii)) carbon and (b­(iii)) phosphorus for disk. Chemical characterization of the cathode material: (c) FTIR, (d) Raman shift, and (e) XRD for LFP, rGO, and the patterned cathodes.

### Electrochemical Performance

With the cathode–electrolyte interface playing a crucial role in the electrochemical performance of the cathode, patterning the active material as they are deposited can have direct influence over the battery output that should be weighed against the deposited pattern to determine its potential for introducing tailorability in cathode design.

We used cyclic voltammetry (CV) at a 0.1 mV s^–1^ scan rate to characterize the electrochemical performance, polarization shifts, and hysteresis of the patterned cathode specimens against a control specimen, i.e., conventional LFP cathode, LFP:PVP:carbon black with a weight ratio of 93:2:5 (Figure 4a). We observed a considerable voltage gap between the disk, ring, and control specimen, which was previously observed in other works,^16^ which was attributed to higher rGO content for improving the cathode performance due to its higher electrical conductivity and percolated network. Cells with either of the cathodes, i.e., ring or disk, exhibited symmetrical peaks—a sign of good reversibility—but also showed distinctive differences. For instance, the disk pattern has slightly sharper peaks and higher peak current than the ring’s, suggesting better reaction kinetics and faster charge transfer because of higher conductivity and more active surface area. The disk cathode specimen also has a slightly smaller difference between oxidation and reduction peaks, i.e., potential separation, indicating reduced polarization compared to the ring. While having ∼80–85% of the peak current and a less efficient charge transfer process, the slightly broader peaks of the ring cathode indicate slower kinetics, which gives it a slight edge under prolonged cycling or at higher currents by reducing side reactions and parasitic losses.

**Figure 4:**
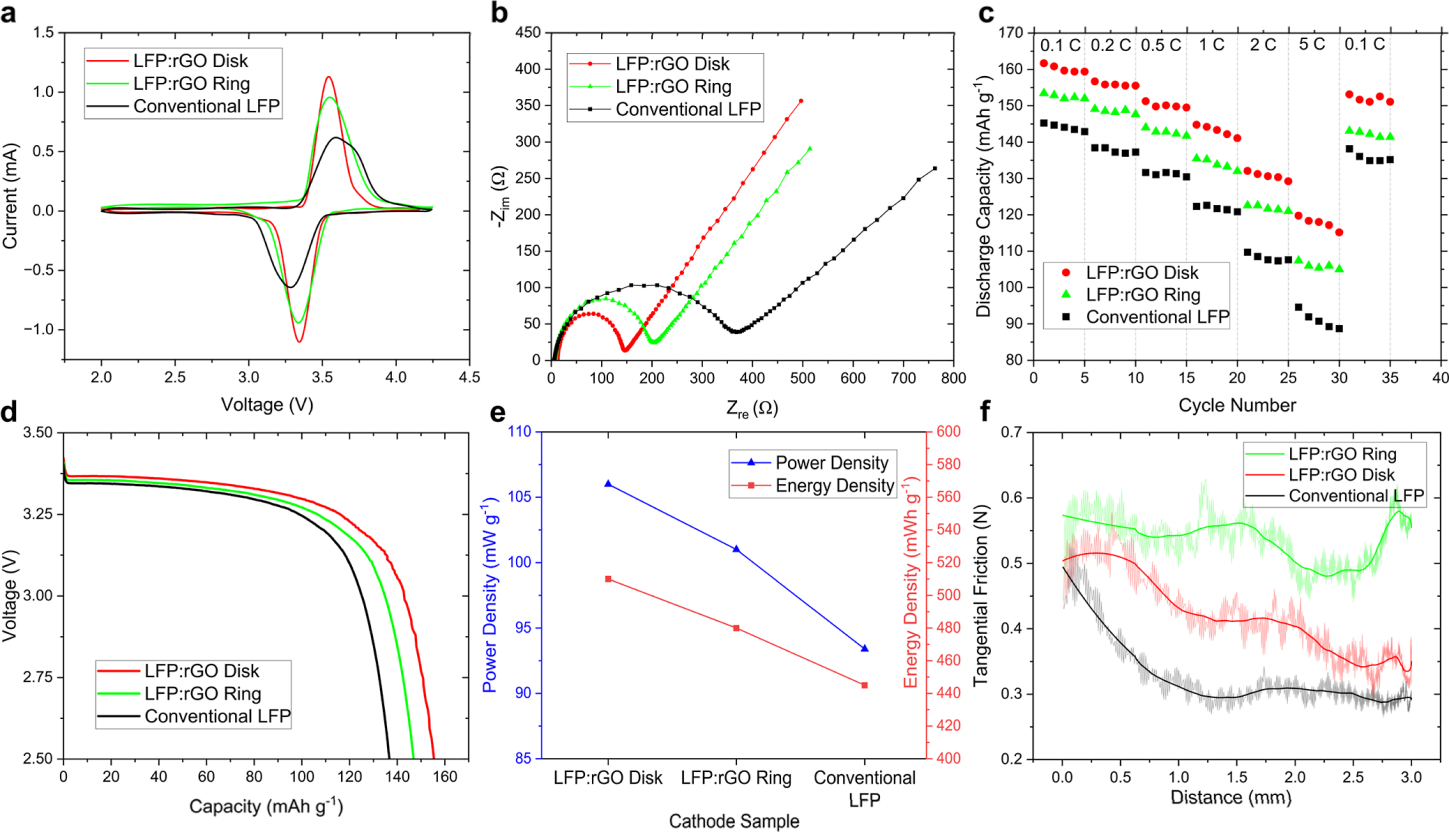
Electrochemical performance comparison of LFP:rGO cathodes with disk and ring patterns: (a) Cyclic voltammetry shows the maximum voltage advantage of the disk pattern over the ring. The advantage of the disk pattern in electrochemical performance is also evident in its low charge transfer resistance shown in the (b) Nyquist plot and the potential to retain a higher charge in (c) discharge capacity retention index compared to rings. (d) Voltage–capacity profiles at 0.2 C across multiple cycles show a higher capacity for the disk pattern than the ring. The electrochemical performance comparison between the patterns is summarized in (e) power density and energy density, where the disk pattern outperforms. The ring pattern shows better interfacial adhesion and mechanical performance, represented by (f) tangential friction force.

We used electrochemical impedance spectroscopy (EIS) to delve further into the charge transfer of the cathode (Figure 4b). The charge transfer resistance (*R*_ct_), which reflects electron transfer kinetics, was approximately 380 Ω for the control specimen—substantially higher than the values of 150 and 220 Ω observed for the disk and ring specimens, respectively. This means despite the disk-patterned cathode outperforming its ring-patterned counterpart, both demonstrate significantly faster charge transfer processes than the control specimen. We tracked the cycling stability and reversible capacity under various C-rates (Figure 4c). While both rGO–LFP cathodes operate at higher discharge capacities than the control conventional Li cathode, their performance begins to diverge more significantly at higher C-rates; the disk specimen starts with a slight advantage of ∼4.5% over the ring at 0.1 C, to a significant margin of ∼9.5% at 5 C. Throughout each cycling process, however, the ring specimen maintained a charge–discharge efficiency consistently above the disk specimen, highlighting its more effective energy retention especially at higher C-rates (96.1% vs 97.7% at 5 C). The presence of a more percolated continuous network in disk patterns is primarily responsible for the superior performance of the disk specimen; however, we hypothesize that the excessive presence of a conductive network (rGO here) can also lead to increased side reactions, resulting in greater capacity deterioration during cycling. Despite this limitation, the disk-patterned cathode demonstrated robust cycling behavior and retained ∼85% capacity after 100 cycles at 1 C, maintaining adhesion and minimizing fluctuations via a percolated conductive rGO network and buffering volume changes potentially via central expansion (Figure S4). We followed the voltage profile during discharge to determine the discharge capacity over the voltage (Figure 4d). The LFP:rGO disk specimen shows a higher discharge capacity and a flatter discharge plateau, indicating more stable operation than 8:1, consistent with the results depicted previously. Still, the well-defined voltage plateau—indicative of Li^+^ intercalation/deintercalation—for both specimens suggests stable performance. Based on the discharge performance results, we calculated the maximum power and energy output, normalized to the weight of the active material, i.e., LFP (see Figure 4e). The data reveal that the all-disk-patterned cathode slightly outperforms all-ring samples, achieving approximately 106 mW g^–1^ and 510 mWh g^–1^.

We used tribometer scratch test results to assess the surface-to-pattern adhesion of the ring and disk cathode materials to the aluminum substrate (Figure 4f). The results depict a ∼25% drop in the tangential friction force with the transition from ring to disk patterns. This indicates that the ring-patterned cathodes exhibit greater interfacial strength/adhesion—reflecting a higher overall structural integrity compared to the disk-patterned structured surface. This is attributed to higher inner and outer surface area of ring patterns, which enhance the interfacial adhesion compared to disks. We also observed a similar trend in tape adhesion results (Figure S2). In conjunction with the scratch test data obtained from the tribometry (Figure S3), depicting a similar trend in intrinsic integrity of the patterns, we hypothesize that the superior internal cohesion, combined with the increased interacting surface area of rings—a thin wall with an average height of three-times higher than the average height of the disk pattern—provides better adhesion at the electrode–substrate interface, leading to improved stability at the interfaces in the ring-patterned cathode.

On a larger scale, these results highlight the electrochemical superiority of the disk-patterned cathode over the ring-patterned design, demonstrating enhanced performance in key metrics such as peak current, discharge capacity, and output power. However, the data also underscore the structural advantages of the ring-patterned cathode, including greater adhesion and a larger interacting surface area, which can improve current substrate–electrode–electrolyte interface integrity. This means a combination of ring and disk patterns, applied to the cathode surface via a specialized spray technique, can achieve a predesigned optimal balance of electrochemical performance, mechanical integrity, and longevity tailored to our specific needs—offering greater design flexibility compared to the rigid product of the traditional slurry method. To evaluate this possibility, we fabricated a combined ring–disk cathode (see Supporting Information for manufacturing details) and tracked its key electrochemical and mechanical properties.

The Nyquist plot (Figure 5a), where the combined ring–disk-patterned cathode (50% ring–50% disk) is compared to the individual ring- and disk-patterned specimens, reveals electrochemical performance closely following that of the previously leading disk specimen (Figure 5a). Particularly, the ring–disk cathode shows improved ion diffusion at the interface (higher Warburg slope) compared with singular ring and disk specimens. We see a similar trend in the capacity–voltage measurements, where the combined specimen matches or outperforms the best-performing singular pattern (disk) specimen (Figure 5b). Moreover, the combined specimen depicts a structural integrity higher than or equal to that of the ring specimen (Figure 5c), which has better interfacial adhesion compared to individual disk’s. The results demonstrate a remarkable synergistic interaction between the disk and ring architectures: the combined cathode demonstrates electrochemical and mechanical performance comparable to, and in some cases exceeding, that of the individual patterns. We hypothesize that the enhanced performance of the combined ring–disk cathode arises from a cooperative interplay between the complementary functionalities of each pattern: the disk regions facilitate efficient electron and ion transport due to higher active material density, while the ring regions enhance interfacial adhesion and mechanical robustness through an increased edge area and stress dissipation. This spatially heterogeneous architecture likely promotes a more uniform charge distribution and mitigates delamination or localized failure, thereby enabling simultaneous optimization of electrochemical and mechanical properties. The attained synergy surpasses a mere balance of properties by enhancing multifunctional behavior not attainable by either pattern alone, provides a viable strategy for future cathode design optimizations, and overcomes the traditional trade-off between electrochemical and mechanical properties.

**Figure 5:**
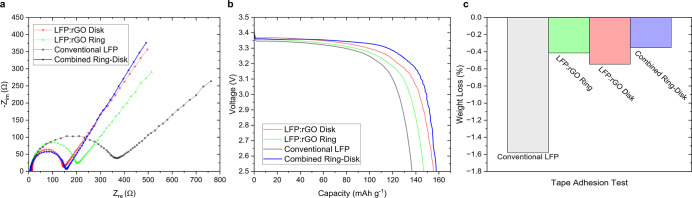
Revealing combined patterns synergy: (a) Nyquist plot and (b) voltage–capacity profile comparing the combined ring–disk-patterned cathodes matching the disk specimen, the best-performing electrochemical specimen (C-rate was set to 0.2 C). (c) Weight loss index from the tape adhesion test, showcasing the combined ring–disk cathodes matches/outperforms the best-performing mechanical specimen, i.e., ring-patterned cathode in structural integrity.

### Simulation Results

Simulations—ranging from microscale molecular dynamics to macroscale numerical methods—have been an essential tool for investigating the fundamental factors affecting lithium-ion battery design and performance.^17^ We performed ab initio molecular dynamics (AIMD) simulations for both LFP:rGO specimens—individual ring and disk patterns—to gain atomic-level insights that govern transport mechanism, lithiation behavior, and structural stability. Despite the inherent limitations in cell size and time and length scales, this approach provides mechanistic insights into how atomic-level processes drive the macroscopic electrochemical and mechanical enhancements observed experimentally. We observed that in the LFP:rGO disk model that constitutes the disk-shaped pattern, lithium ions diffuse faster due to the formation of well-defined transport channels. These channels are primarily created by the interaction between the rGO and phosphate ions (PO_4_^3–^), and they are represented in sky blue color in Figure 6a. These phosphate ions (PO_4_^3–^) form a tetrahedral structure, stabilizing lithium transport and reducing energy barriers along the Li^+^ diffusion pathways. Additionally, the layer-by-layer diffusion mechanism observed in these structures enhances ionic mobility, meaning that lithium can move more easily between layers without facing significant resistance. However, although the presence of many diffusion channels helps improve the initial performance of the material, it also introduces potential trapping sites for lithium ions. Some Li^+^ ions can become stuck inside these channels, especially if the structure slightly deforms over time. This lithium trapping effect can lead to capacity fading, where the battery loses its ability to store and release lithium effectively during repeated charge and discharge cycles. This explains why the disk-shaped patterns initially exhibit higher electrochemical performance but tend to degrade faster over extended cycling. On the other hand, the ring models that contain less rGO show a different lithium diffusion behavior (Figure 6b), where the structure lacks the extensive sky-blue transport pathways seen in the disk model. As a result, lithium ions (purple) move through shorter but more confined pathways, leading to a more controlled diffusion process that is a major contributor to the enhanced stability observed in the ring-like patterned cathodes.

**Figure 6:**
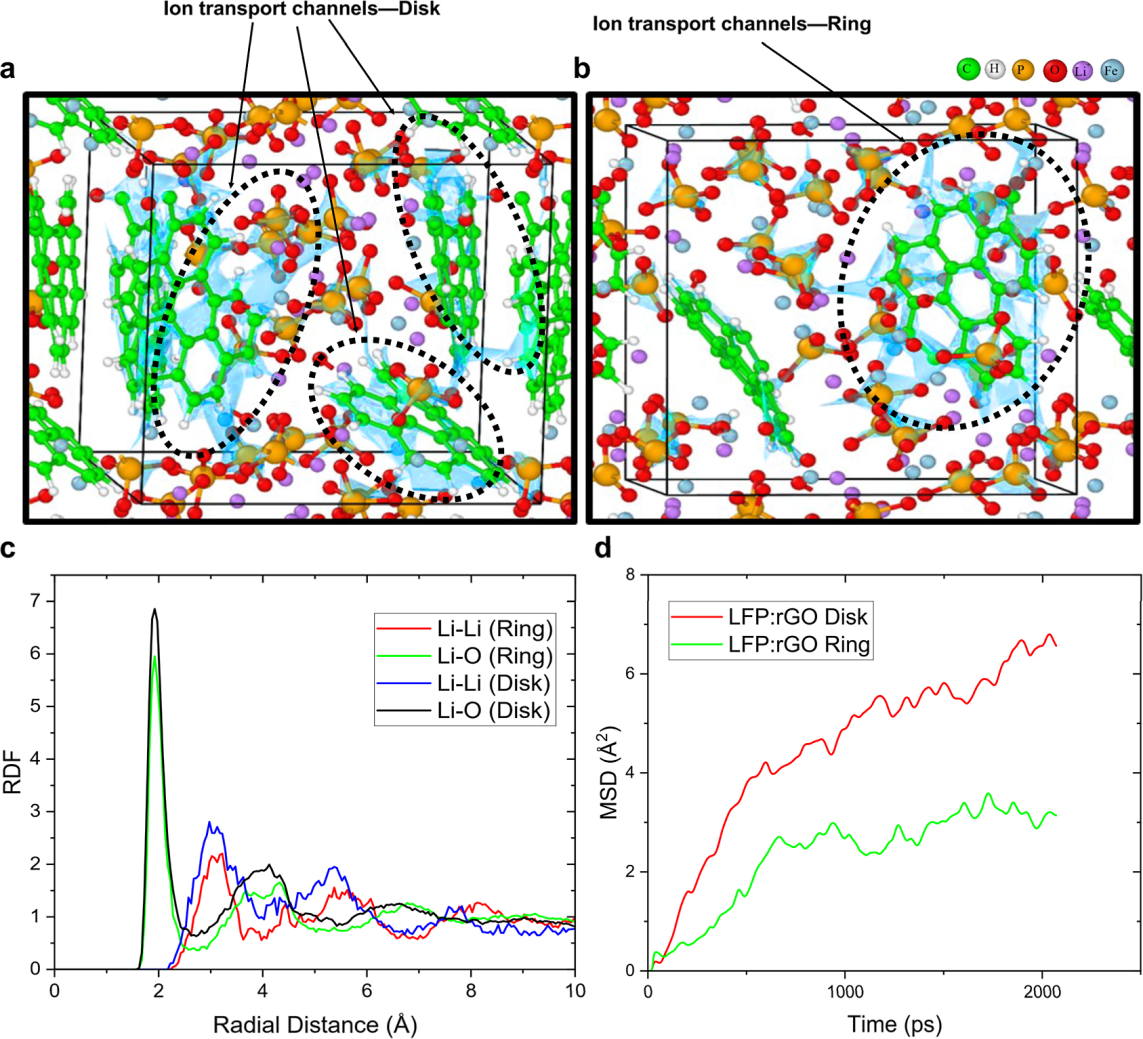
AIMD models of (a) disk and (b) ring. The diffusion channels are highlighted in sky blue, centered around the rGO nanoparticles. The pathways form where rGO nanoparticles interact with phosphate ions (PO_4_^3–^), depicting a low-energy planar diffusion path where Li ion has a higher mobility due to the relatively weaker interactions. AIMD results: (c) RDF index characterizes the disk model with stronger peaks for the Li–Li pair, signaling a more compact presence of Li ions within the formed pathways which also provide higher mobility, as depicted in the (d) MSD results, ultimately leading to superior electrochemical performance of the disk specimen.

We analyzed the Radial Distribution Function (RDF) index to track the relative positioning of Li–Li and Li–O pairs in both the ring and disk models during the last 1 ps of the AIMD simulations (Figure 6c). In both disk and ring pattern models, the first Li–Li and Li–O peaks appear at 3.1 Å and 2 Å, respectively. The​ difference becomes apparent as we compare the strength of the peaks: for the Li-Li pair distance distribution, the disk model shows a higher correlation (depicted by a taller peak) at both ∼3.1 Å and 5.3 Å, signifying that lithium ions are more likely to reside closer to each other in small groups and follow more defined paths. This could explain why lithium moves faster in the disk-shaped patterns but also why some lithium might get stuck over time. This means the ring pattern model is expected to have lithium ions spread out more evenly and fewer lithium ions get trapped in specific locations. RDF analysis also confirms that lithium interacts with oxygen from phosphate ions (PO_4_^3–^), forming layered diffusion paths. The disk model shows stronger peaks in the Li–O pair, meaning a stronger interaction in the disk model that might pose as potential traps during the charge/discharge process. This matches what we saw in the AIMD simulations, where lithium moves through well-organized pathways in the disk model but follows shorter, more confined paths in the ring model.

To further analyze lithium-ion movement within the LFP:rGO composite, we calculated the Mean Square Displacement (MSD) of Li^+^ ions for both disk and ring pattern models (Figure 6d). The results show that the Li^+^ cations have a greater displacement in the disk pattern model compared to the ring pattern model. By averaging the MSD values over the last 0.5 ps, we obtained 6.1 Å^2^ for the disk model, corresponding to a diffusion coefficient of 2.03 × 10^–9^ m^2^ s^–1^, nearly twice that of the ring pattern model, which was 1.07 × 10^–9^ m^2^ s^–1^ for an average MSD of 3.2 Å^2^. These results confirm that lithium diffuses more rapidly in the disk model, which is consistent with the presence of well-defined diffusion channels observed in the AIMD simulations. The disk-patterned cathode possesses high electrical and ionic conductivity that delivers higher power and capacity compared to the other specimens. The situation is quite different in the ring-patterned cathode, where lithium ion diffusion is somewhat slower; while the lower rGO creates fewer channels, Li^+^ ions are less likely to get trapped (and become “dead lithium”—lithium that becomes inactive and no longer contributes to battery performance); this means the ring-patterned cathodes can potentially preserve a higher portion of their capacity for longer.

## Conclusions

Traditional cathode fabrication relies on planar, two-dimensional structures, often leading to trade-offs between capacity, power, interfacial adhesion, and mechanical strength. This study introduces incorporating three-dimensional surface patterning into the cathode design. By precisely controlling the weight ratio of LFP and rGO in the dispersion being deposited onto an aluminum substrate, we achieved two distinct morphologies for manufacturing cathodes: disk-shaped and ring-shaped patterns. We demonstrated that these variations align with distinct shifts in the electrochemical and mechanical properties. The disk-patterned specimen exhibits superior electrochemical performance, i.e., higher discharge capacity, better kinetics, more stable voltage, lower charge transfer resistance, and higher energy and power density compared to ring counterparts. Meanwhile, we observed that the ring-patterned cathode exhibits superior mechanical properties, i.e., higher interfacial strength, compared with its disk-patterned counterpart. When cathodes are assembled from repeatable, spatially controlled mixtures of disk- or ring-patterned spray deposits—arranged laterally or vertically—the resulting structures amplify specific properties, such as electrochemical or mechanical properties. In addition to intrinsic materials properties, we showed that these advantages also emerged from morphological shapes, where disk provided a more connected percolated network for ion and electron transport and ring’s toroidal surfaces result in a larger, more cohesive interacting surface area that enhances the electrode–electrolyte interfacial interactions. Notably, an architecture comprising a combined disk–ring pattern demonstrates a pronounced synergistic effect, achieving electrochemical behavior comparable to the disk-patterned specimen while maintaining or exceeding the mechanical performance of the ring-patterned design. This emergent functionality is potentially arising from the spatial integration of distinct morphological domains, where the disk regions facilitate localized charge transport and energy storage and buffer volume changes via central expansion, while the ring regions enhance interfacial adhesion and mitigate stress accumulation. At the molecular level, our MD simulations reveal that although Li^+^ ions navigate the cathode material in disk patterns more easily because of higher rGO content, they are more prone to being trapped, potentially degrading the long-term performance compared to the ring-patterned cathode. These findings highlight the potential of geometry-guided morphology-architecture engineering where strategically combining patterns of different geometries allows reconciling properties traditionally subject to engineering trade-offs, enabling more versatile design for high-performance cathodes.

## Methods

### Material Specification

LFP was procured from Landt International Inc. with a particle size distribution median (D50) of 1.30 μm and a surface area (BET) of 13.36 m^2^ g^–1^. Reduced graphene oxide was purchased from ACS Material with a D50 of 20 μm and a BET of 390 m^2^ g^–1^.

### Preparation Method

The dispersion intended for spraying was prepared by using probe sonication. In the first stage, rGO was added to DMF and sonicated for 10 min. After that, LFP was added to the dispersion, and the mixture underwent an additional 20 min of sonication under the same conditions.

The spray-deposition process was conducted inside an argon-filled glove bag to minimize humidity contamination of the active material. Nitrogen gas was used as the carrier gas for spraying, ensuring a controlled deposition environment that prevented the oxidation and moisture-induced degradation of the electrode components. The prepared dispersion was loaded into a high-precision spray gun that was calibrated to deliver a spray pressure of 20 MPa. The distance between the nozzle and the substrate was maintained at 15 cm, and the spray gun was held at a 45° angle to the substrate to ensure uniform coverage without causing the dispersion to drip. Short pauses between each spray round allowed for the evaporation of the DMF solvent, preventing droplet agglomeration and ensuring an even coating across the surface of the substrate. After multiple rounds of spray deposition, the total active material mass deposited on the substrate was measured (Tables S1 and S2 and Figure S1).

Coin cells were assembled in an argon-filled glovebox. The LFP/rGO-coated Al cathode, Celgard separator, and lithium metal anode were stacked in their respective order inside the case. The separator and electrodes were wetted with the LiPF_6_/EC-DMC electrolyte (1.0 M lithium hexafluorophosphate (LiPF_6_) in a 1:1 mixture of ethylene carbonate (EC) and dimethyl carbonate (DMC)). To ensure contact, spacers and a spring were placed before sealing the cell using a crimper.

### Surface Morphology Characterization

The surface features and morphology of the prepared specimens were assessed using Bruker DektakXT Surface Profiler, VR-5000 3D Optical Profiler, and scanning electron microscopy (SEM, Tescan FERA-3). The chemical composition and functional groups of the electrode material were analyzed using Fourier Transform Infrared Spectroscopy (FTIR) with an attenuated total reflectance (ATR) module (Bruker ALPHA II FTIR). The crystalline structure of the electrode material was analyzed using X-ray diffraction (D8 Advance ECO, Bruker, Germany). Raman spectroscopy was conducted using an i-Raman Plus 532 nm Raman Spectrometer (B&W Tek, Metrohm, USA).

### Mechanical Interfacial Strength Characterization

Tribological analysis was performed using an Anton Paar TRB Tribometer (to measure the tangential friction coefficient) with a WC pin moving at a speed of 1 cm s^–1^ with a normal load of 1 N for a scratch length of 3 mm. We performed a scratch test on the same surfaces using a Hysitron TI 950 Triboindenter, with a constant normal force of 20 mN over a scratch length of 250 μm. We also evaluated the adhesion strength of the active material to the substrate by measuring the weight loss in the tape adhesion test. The test was conducted with a 200 g weight for the first eight cycles, followed by 1540 and 6540 g for the second and third eight-cycle stages, respectively.

### Electrochemical Measurements

Electrochemical measurements were conducted using a potentiostat (Interface 1010E Gamry). Both cyclic voltammetry (CV) tests and galvanostatic charge/discharge (GCD) experiments were performed within a voltage range of 2 to 4.25 V (vs Li/Li^+^). For CV tests, a scan rate of 0.1 mV s^–1^ was maintained, and the tests were run for 10 cycles. Specific capacity measurement was performed with a multichannel Gamry battery system with varying C-rates of 0.1 C, 0.2 C, 0.5 C, 1 C, 2 C, and 5 C to evaluate their specific capacity under different current densities (C-rate of 0.2 C was used to obtain capacity measurements). Each coin cell was charged to a cutoff voltage of ∼4.2 V at the specified C-rate and then discharged to a lower cutoff voltage of ∼2.5 V at the same rate. We used the weight of the active material (LFP) to normalize the power and energy to minimize the impact of variations of weight on the results. Electrochemical impedance spectroscopy (EIS) was performed in the frequency range from 10,000 to 0.01 Hz under an alternating current with a 10 mV amplitude (Interface 1010E, GAMRY Instruments). The impedance data were fitted by using the GAMRY Echem Analyst program.

### Simulations

All calculations were performed under periodic boundary conditions in the *NVT* ensemble at a temperature of 300 K. The Nosé thermostat^18^ was used to control temperature fluctuations, with a mass parameter of 0.5. To enable a longer time step of 1 fs, the hydrogen mass was adjusted to the tritium mass. The energy cutoff was set to 400 eV, and Gaussian smearing was applied with a width of 0.05 eV. For Brillouin zone sampling, the Monkhorst–Pack^19^ method was used with a *k*-point density of 1 × 1 × 1. The Perdew–Burke–Ernzerhof (PBE)^20^ generalized gradient approximation (GGA) was employed for the exchange–correlation functional, and the Projector Augmented Wave (PAW)^21^ method was used to describe core–electron interactions. Each ab initio molecular dynamics (AIMD) simulation was run for at least two picoseconds to ensure system stabilization. The energy oscillations were monitored, and the system was considered stabilized when the average energy fluctuations were ≤0.01 eV per atom.

## Supplementary Material



## Notes

Detailed methodology, tables of composition for the prepared dispersions and cathode specimens, schematic portrayal of the electrode manufacturing process, and plots of tape adhesion and scratch test results and long-term discharge data for the LFP:rGO disk cathode mentioned in the main text (PDF)

## References

[1] Nazri, G.-A. ; Pistoia, G. Lithium Batteries: Science and Technology; Springer Science & Business Media, 2008.

[2] Xie G., Zhu H.-J., Liu X.-M., Yang H. (2013). A core–shell
LiFePO4/C nanocomposite prepared via a sol–gel method assisted
by citric acid. J. Alloys Compd..

[3] Padhi A. K., Nanjundaswamy K. S., Goodenough J. B. (1997). Phospho-olivines as positive-electrode
materials for rechargeable lithium batteries. J. Electrochem. Soc..

[4] Xu D., Wang P., Shen B. (2016). Synthesis
and characterization
of sulfur-doped carbon decorated LiFePO4 nanocomposite as high performance
cathode material for lithium-ion batteries. Ceram. Int..

[5] Yang L., Yang K., Zheng J., Xu K., Amine K., Pan F. (2020). Harnessing the surface structure
to enable high-performance cathode materials for lithium-ion batteries. Chem. Soc. Rev..

[6] Song W., Liu J., You L., Wang S., Zhou Q., Gao Y., Yin R., Xu W., Guo Z. (2019). Re-synthesis of nano-structured LiFePO4/graphene composite
derived
from spent lithium-ion battery for booming electric vehicle application. J. Power Sources.

[7] Zhang Y., Huo Q.-y., Du P.-p., Wang L.-z., Zhang A.-q., Song Y.-h., Lv Y., Li G.-y. (2012). Advances
in new cathode material LiFePO4 for lithium-ion batteries. Synth. Met..

[8] Wu X.-L., Guo Y.-G., Su J., Xiong J.-W., Zhang Y.-L., Wan L.-J. (2013). Carbon-Nanotube-Decorated
Nano-LiFePO4
@C Cathode Material with Superior High-Rate and Low-Temperature Performances
for Lithium-Ion Batteries. Adv. Energy Mater..

[9] Ho Lee S., Grant P. S. (2023). Spray fabrication of additive-free
electrodes for advanced Lithium-Ion storage technologies. J. Colloid Interface Sci..

[10] Wang H., Li J., Miao Z., Huang K., Liao Y., Xu X., Meng J., Li Z., Huang Y. (2023). Hierarchical electrode architecture enabling ultrahigh-capacity
LiFePO4 cathodes with low tortuosity. ACS Appl.
Mater. Interfaces.

[11] Shariatnia S., Kaynan O., Jarrahbashi D., Asadi A. (2022). Engineering bottom-up
fabrication of functional multi-material nanostructures created through
evaporation-induced self-assembly of nanocolloidal droplets. MRS Commun..

[12] Kaynan O., Hosseini E., Zakertabrizi M., Motta De Castro E., Pérez L. M., Jarrahbashi D., Asadi A. (2023). Multifunctionality
through Embedding Patterned Nanostructures in High-Performance Composites. Adv. Mater..

[13] Shariatnia S., Asadi A., Jarrahbashi D. (2021). Experimental
analysis of supercritical-assisted
atomization. Phys. Fluids.

[14] Shariatnia S., Kaynan O., Jarrahbashi D., Asadi A. (2022). Engineering bottom-up
fabrication of functional multi-material nanostructures created through
evaporation-induced self-assembly of nanocolloidal droplets. MRS Commun..

[15] Shariatnia S., Zakertabrizi M., Hosseini E., Song K., Jarrahbashi D., Asadi A. (2023). Engineering Multimaterial Nanostructured
Deposits by the Amphiphilicity
Degree and Intermolecular Forces. Adv. Mater.
Technol..

[16] Muchuweni E., Mombeshora E. T., Muiva C. M., Sathiaraj T. S. (2024). Towards high-performance lithium-ion
batteries by introducing graphene-based materials into LiFePO4 cathodes:
A review. NanoTrends.

[17] Hasani E., Torabi F., Salavati-Zadeh A. (2024). Electrochemical
simulation of lithium-ion batteries: a novel computational approach
for optimizing performance. Hydrogen, Fuel Cell
Energy Storage.

[18] Nosé S. (1984). A unified formulation
of the constant
temperature molecular dynamics methods. J. Chem.
Phys..

[19] Monkhorst H. J., Pack J. D. (1976). Special points for Brillouin-zone
integrations. Phys. Rev. B.

[20] Perdew J. P., Burke K., Ernzerhof M. (1996). Generalized
Gradient Approximation
Made Simple. Phys. Rev. Lett..

[21] Kresse G., Joubert D. (1999). From ultrasoft pseudopotentials
to the projector augmented-wave
method. Phys. Rev. B.

