# Supplementary material for: Patterned Nanostructures on Cathodes: A Pathway to Stronger, High-Energy, High-Power Li-Ion Batteries
Source: ACS Nano. 2025 Nov 3;19(45):38970–80. doi: 10.1021/acsnano.5c07146 (PMC12632169; doi:10.1021/acsnano.5c07146)
Supplement: Supplementary file 1 [file nn5c07146_si_001.pdf]

Supporting Information

Patterned Nanostructures on Cathodes: A Pathway to Stronger, High-Energy, High-Power Li-Ion Batteries

Mohammad Zakertabrizi[a, †], Farshad Bozorgmehrian[b, †], Myunghwan Jeong[c], Ehsan Hosseini[a], Victor Ponce[c], Hamed Fallahi[a], Saeed Bahadorikhalili[d], Hadi Nasrabadi[d], Dorrin Jarrahbashi[a], Homero Castaneda[c], Amir Asadi[b,c*]

[a] Department of Mechanical Engineering, Texas A&M University, College Station, Texas 77843, United States

[b] Department of Engineering Technology and Industrial Distribution, Texas A&M University, College Station, Texas 77843-3367, United States

[c] Department of Materials Science and Engineering, Texas A&M University, College Station, Texas 77843-3367, United States

[d] Department of Petroleum Engineering, Texas A&M University, College Station, Texas 77843-3367, United States

*Corresponding author: amir.asadi@tamu.edu

[†]Authors contributed equally to this work.

Material Specification

In this study, rGO from ACS Material was synthesized using a chemical reduction method with hydrazine (N_2H_2) as the reducing agent. The resulting material exhibited a distinctive brownish-grey coloration, indicative of successful reduction. The rGO used had an average thickness of approximately 1 nm, falling within the nanometer range, making it an excellent candidate for applications requiring high surface area and conductivity. The rGO samples had a monolayer diameter distribution ranging between 0.5 and 10 μm, with a monolayer rate exceeding 90%, ensuring that most of the rGO was present as single layers, maximizing its conductive properties. The BET surface area of the material was measured at approximately 180 $m^2\ g^{-1}$, indicative of its high surface area and thus its suitability for energy storage applications. The carbon content of the material exceeded 82 wt.%, further validating the effectiveness of the reduction process. The rGO's conductivity, recorded at over 500 S/m, reinforced its potential as a material for high-performance electrochemical applications, particularly in battery systems, where conductivity is paramount for efficient charge/discharge cycles.

LFP sourced from Landt International Inc. was also used in this study. LFP, recognized for its stable electrochemical properties, had a particle size distribution median (D50) of 1.302 μm, making it ideal for use in lithium-ion battery applications. The specific surface area, determined by the BET method, was measured at 13.36 m^2/g, enhancing its interaction with conductive additives like rGO. LFP's density of 1.14 g/cm^3 ensured that it packed tightly in the electrode structure, contributing to a higher energy density. The electrochemical performance of LFP was further assessed, with a measured discharge capacity of 153 mAh/g at a 0.1C rate under test conditions of 25°C, within a voltage range of 2.0–4.2 V. These properties collectively demonstrate LFP's role in optimizing the overall energy storage capacity and durability of the battery systems under investigation.

Preparation Method

Sonication was performed in two stages to ensure the uniformity of the mixture. In the first stage, rGO was added to DMF in the beaker and sonicated at 40% amplitude for 10 minutes using a probe sonicator, with the pulse option set to 30 seconds on and 30 seconds off. This pulsing method helped avoid excessive heat buildup during the process, allowing for more effective dispersion of the rGO particles throughout the dispersion. After the initial sonication, LFP was added, and the mixture underwent an additional 20 minutes of sonication under the same conditions. The two-stage sonication process was essential to prevent particle agglomeration, which could have negatively impacted the uniformity of the spray deposition (Table S1).

The spray-deposition process was conducted inside a nitrogen-filled glove bag to minimize humidity contamination of the active material. Nitrogen gas was also used as the carrier gas for supercritical

spraying, ensuring a controlled deposition environment that prevented oxidation and moisture-induced degradation of the electrode components. The dispersion was loaded into a high-precision spray gun, which was calibrated to deliver a spray pressure of 20 MPa. The distance between the nozzle and the substrate was maintained at 15 cm, and the spray gun was held at a 45° angle to the substrate to ensure uniform coverage without causing the dispersion to drip. Short pauses between each spray round allowed for the evaporation of the DMF solvent, preventing droplet agglomeration and ensuring an even coating across the surface of the substrate.

After multiple rounds of spray deposition, the total active material mass deposited on the substrate was measured. The precise control of deposition mass and density was critical to the consistency of the electrode's performance during electrochemical testing. Figure S1 shows a graphical view of the process, and Table S2 provides details of each case composition. We fabricated the combined ring-disk specimen using a modified method. After applying two consecutive sprays from the ring dispersion, we followed with two sprays from the disk dispersion, repeating this alternating pattern until achieving the same total number of sprays.

Coin Cell Fabrication

The assembled electrodes were incorporated into coin cells within a glove box, which maintained an argon atmosphere to prevent exposure to air or moisture that could degrade the components. The machined aluminum substrates, now coated with LFP/rGO, were positioned as the cathodes. A Celgard separator was placed over the cathode to avoid short-circuiting within the cell.

To ensure optimal ionic conductivity, electrolyte solution (1.0 M lithium hexafluorophosphate ($LiPF_6$) in a 1:1 mixture of ethylene carbonate (EC) and dimethyl carbonate (DMC)) was added to the separator. The lithium metal counter electrode was then placed on top, followed by stainless steel spacers and a spring. The cell assembly was completed by sealing the structure with the anode side and crimping the cell using a compact crimping machine, applying sufficient force to ensure the cell was sealed but without over-crimping, which could damage the structure.

Surface Characterization

The surface morphology of the electrode was examined using a Tescan FERA-3 Model GMH Focused Ion Beam Scanning Electron Microscope (FIB-SEM). High-resolution SEM images were obtained at an accelerating voltage of 20 kV to analyze the microstructural features and surface characteristics of the electrode. In addition to morphological assessment, energy-dispersive X-ray spectroscopy (EDS) was performed to determine the elemental composition and spatial distribution of elements across the electrode

surface. The EDS analysis provided qualitative and semi-quantitative data, enabling the visualization of elemental distribution maps for key constituents of the electrode material.

Fourier Transform Infrared Spectroscopy

The chemical composition and functional groups of the electrode material were analyzed using Fourier Transform Infrared Spectroscopy (FTIR) with an attenuated total reflectance (ATR) module. Measurements were conducted using a Bruker ALPHA II FTIR spectrometer, which allows for non-destructive surface analysis without extensive sample preparation. The spectra were recorded within the relevant wavenumber range to identify characteristic vibrational modes corresponding to the functional groups present in the electrode material. ATR-FTIR analysis provided insights into molecular interactions and potential chemical modifications occurring on the electrode surface.

X-ray Diffraction (XRD)

The crystalline structure of the electrode material was analyzed using an X-ray diffractometer (D8 Advance ECO, Bruker, Germany). The instrument was operated with Cu Kα radiation (λ = 1.5406 Å) at 40 kV and 25 mA. Diffraction patterns were recorded over a 2θ range of 4° to 74°, allowing for phase identification and structural characterization of the material. The obtained XRD data provided insights into the crystallinity, phase composition, and potential structural modifications of the electrode.

Raman Spectroscopy

Raman spectroscopy was conducted using an i-Raman Plus 532 nm Raman Spectrometer (B&W Tek, Metrohm, USA) to analyze the vibrational modes and molecular structure of the electrode material. The measurements provided insights into the chemical bonding, phase composition, and structural integrity of the material, allowing for the identification of characteristic peaks associated with its components.

3D Optical Profilometry

The surface topography and roughness of the electrode were characterized using a VR-5000 3D Optical Profiler at 160× magnification. This non-contact measurement system utilizes structured light projection and double-telecentric lenses to minimize distortion and ensure high-precision data acquisition. Height variations across the electrode surface were captured through optical triangulation, with a high-accuracy CMOS sensor enabling detailed 3D imaging. The profiler provided quantitative data on surface roughness, profile variations, and topographical features, offering insights into the morphological characteristics of the electrode.

Surface Profilometry

The surface profile of the electrode was analyzed using a Bruker DektakXT Surface Profiler, a stylus-based contact measurement system designed for high-precision topographical mapping. The profiler was operated with a stylus force set between 1 mg and 2 mg to ensure accurate surface tracing while minimizing potential surface deformation. The obtained profile data enabled the identification of ring and disk patterns formed by droplet deposition on the electrode surface, providing insights into the uniformity and distribution of the material.

Tribometer

Tribological testing was conducted using an Anton Paar TRB Tribometer (Pin on Disk configuration) to measure the tangential friction coefficient of the cathodes. The test utilized a WC pin and was performed with linear motion tangential force at a speed of 1 cm s^{-1}. A normal load of 1 N was applied, and the scratch length was set to 3 mm. The output of this test was the tangential friction coefficient, which was analyzed to assess the material's wear resistance and frictional behavior.

Tape Adhesion Test

To evaluate the adhesion strength of the active material to the substrate, a tape adhesion test was conducted using a controlled weight application method. Each electrode was carefully placed on a strip of paper tape, and a precise weight was applied to ensure uniform pressure across samples. The electrodes were then detached from the tape, and the weight of each electrode was measured before and after detachment to quantify the material loss. To maintain consistency in applied pressure, a 200 g weight was used for the first seven adhesion cycles. Subsequently, the applied weight was increased to 1540 g for cycles eight and nine, followed by 6540 g for the tenth cycle. This stepwise increase in pressure was implemented to assess the progressive impact on material detachment, as initial weight cycles resulted in minimal material loss, necessitating higher applied forces for further evaluation. The results, brought in Figure S2, depict a faster removal rate for the disk specimen. This shows the higher surface-to-pattern cohesion of the ring pattern.

Nano Indenter

The scratch test was performed on the cathodes using a Hysitron TI 950 Triboindenter. A constant normal force of 20 mN was applied over a scratch length of 250 micrometers. The output of the test was the friction coefficient, which was analyzed to evaluate the material's resistance to surface deformation and wear. The results, presented in Figure S3, indicate that the ring pattern exhibits a higher friction coefficient. This suggests that ring patterns possess greater internal cohesion compared to disk patterns.

Electrochemical Measurements

The electrochemical performance of the fabricated cathodes was evaluated using a Gamry Interface 1010E potentiostat and a multi-channel Gamry battery testing system. These measurements were conducted to characterize the electrochemical properties, including cyclic voltammetry (CV) and galvanostatic charge/discharge (GCD) behavior, across a voltage range of 2.0 V to 4.25 V (vs. Li/Li^+). The comprehensive testing protocol aimed to assess the specific capacity, rate capability, and charge/discharge efficiency of the cathodes under varying operational conditions. The electrochemical impedance spectroscopy (EIS) of the batteries was performed in the frequency range from 10000 Hz to 0.01 Hz under an alternating current (AC) with a 10-mV amplitude (Interface 1010E, GAMRY Instruments). The impedance data were fitted using the GAMRY Echem Analyst program. We evaluated the cycling stability of the LFP:rGO disk electrode at a 1C current rate as a representative measure of stability of the new cathode design. The cathode retained ~85% of its initial discharge capacity after 100 cycles, depicting the rGO framework as a highly conductive and mechanically robust structure that effectively buffered the volumetric changes of the active material and maintained continuous electron and ion transport pathways throughout the cyclability test (Figure S4).

Simulations

All calculations were performed under periodic boundary conditions in the NVT ensemble at a temperature of 300 K. The Nosé thermostat was used to control temperature fluctuations, with a mass parameter of 0.5.[1, 2] To enable a longer time step of 1 femtosecond (fs), the hydrogen mass was adjusted to the tritium mass. The energy cutoff was set to 400 eV, and Gaussian smearing was applied with a width of 0.05 eV. For Brillouin zone sampling, the Monkhorst–Pack method was used with a k-point density of 1 × 1 × 1.[3] The Perdew–Burke–Ernzerhof (PBE) generalized gradient approximation (GGA) was employed for the exchange-correlation functional, and the Projector Augmented Wave (PAW) method was used to describe core-electron interactions.[4, 5] Each AIMD simulation was run for at least two picoseconds (ps) to ensure system stabilization. The energy oscillations were monitored, and the system was considered stabilized when the average energy fluctuations were ≤ 0.01 eV per atom. The AIMD simulations were designed to study the lithiation behavior of the LFP-rGO composite systems at 3:1 and 8:1 ratios, focusing on how the disk-shaped and ring-shaped patterns influence the diffusion of lithium ions (Li^+) and the overall structural stability of the cathode materials. By analyzing the atomic trajectories and atomic geometries, we aimed to correlate the observed electrochemical performance with the underlying atomic-scale mechanisms.

Using the Einstein Relation for Diffusion, $D = \frac{MSD(t)}{2dt}$, where $d = 3$ is the number of dimensions and $t = 0.5\ ps$, we calculated the diffusion coefficients. To quantify the interaction strength between lithium

and the electrode materials, we computed the binding energy (De) using the total energy values obtained from first-principles calculations. The total energy for isolated Li^+, rGO, and LFP and their respective combined systems (rGO+Li and Li+LFP) are listed in Table S3.

The binding energy (D_e was calculated as: $D_e = E_{isolated\ atoms} - E_{combined\ system}$ where higher values indicate stronger interactions. The results show that lithium exhibits a significantly stronger binding affinity to LFP (18.326 eV) compared to rGO (1.740 eV). This suggests that lithium is more stable within the LFP structure, while its interaction with rGO is relatively weaker. These differences in binding energy may influence the lithium diffusion dynamics and overall electrochemical performance of the composite material.

Supplementary Tables

Table S1. Composition of dispersions.

Composition Pattern	Disk	Ring
Ratio (LFP:rGO)	3:1	8:1
Weight Ratio in 25 mL DMF	1.5%	1.5%
LFP (g)	0.2812	0.3334
rGO(g)	0.0937	0.0416
Total (g)	0.375	0.375

Table S2. Cathode's information.

No.	Pattern	Spray Times	Initial Weight (mg)	Final Weight (mg)	Deposited Material (mg)	Active Material (mg)
1	Ring	30	586.50	588.55	2.050	1.820
2	Disk	30	591.40	593.75	2.350	1.760
3	Combined Ring-Disk	30	600.10	602.50	2.400	1.966
4	Conventional Cathode	Slurry Casting	584.20	586.22	2.020	2.020

Table S3. Battery components energy and Dissociation energy (D_e).

System	Total Energy (Hartree)	Binding Energy, D_e(eV)
Li^+	-7.280108	
rGO	-1034.156521	
LFP	-641.870148	
rGO+Li	-1041.500577	1.740
Li+LFP	-649.823727	18.326

Supplementary Figures

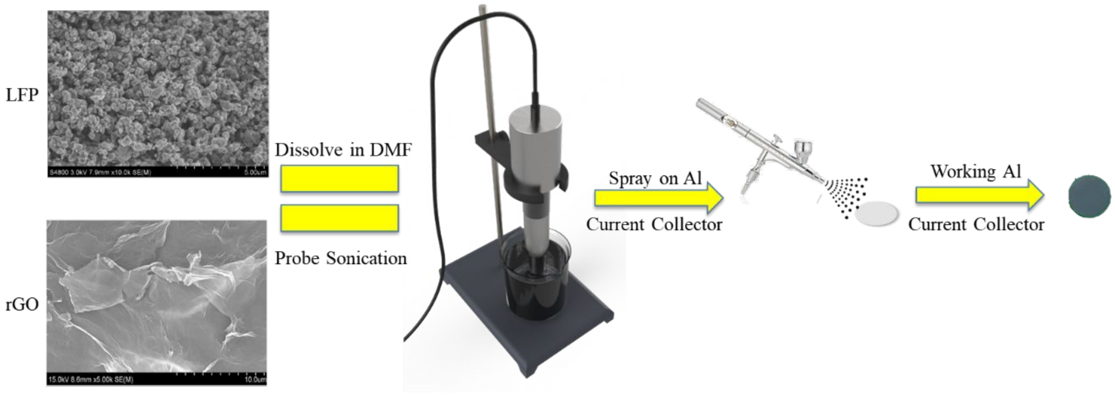

Figure S1. Graphical view of electrode making process using in-house spray method.

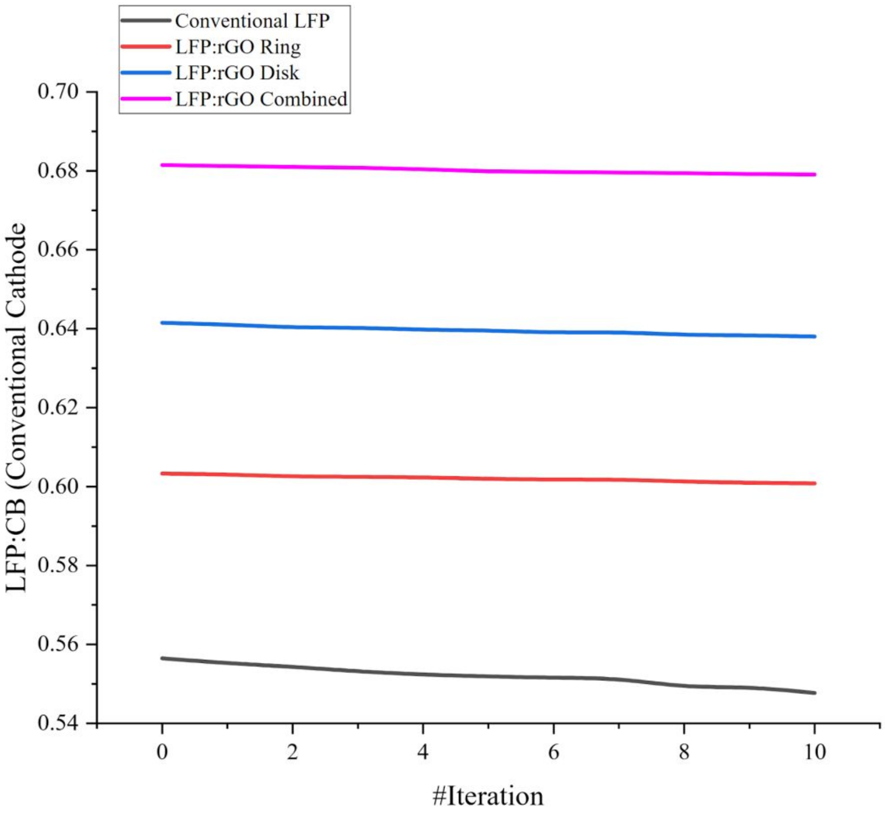

Equation	y = a + b*x
Plot	LFP:rGO Combined
Weight	No Weighting
Intercept	0.68143 ± 8.17424E-5
Slope	-2.53636E-4 ± 1.3817E
Residual Sum of Squares	1.89E-7
Pearson's r	-0.98691
R-Square (COD)	0.97399
Adj. R-Square	0.9711

Equation	y = a + b*x
Plot	LFP:rGO Disk
Weight	No Weighting
Intercept	0.64126 ± 7.72965E-5
Slope	-3.37273E-4 ± 1.30655E
Residual Sum of Squares	1.69E-7
Pearson's r	-0.99331
R-Square (COD)	0.98667
Adj. R-Square	0.98519

Equation	y = a + b*x
Plot	LFP:rGO Ring
Weight	No Weighting
Intercept	0.60323 ± 4.51759E-5
Slope	-2.40909E-4 ± 7.63612E
Residual Sum of Squares	5.77273E-8
Pearson's r	-0.99551
R-Square (COD)	0.99104
Adj. R-Square	0.99004

Equation	y = a + b*x
Plot	LFP:CB (Conventional Cat hode
Weight	No Weighting
Intercept	0.55607 ± 2.19596E-4
Slope	-8.05455E-4 ± 3.71184E-
Residual Sum of Square	1.364E-6
Pearson's r	-0.99058
R-Square (COD)	0.98124
Adj. R-Square	0.97916

Figure S2. Tape Adhesion Test results.

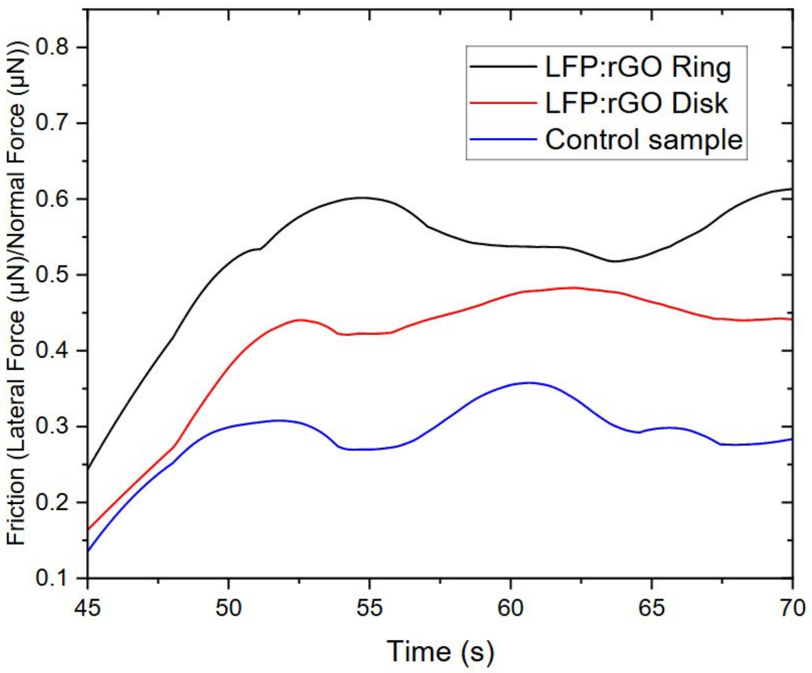

Figure S3. Scratch test results.

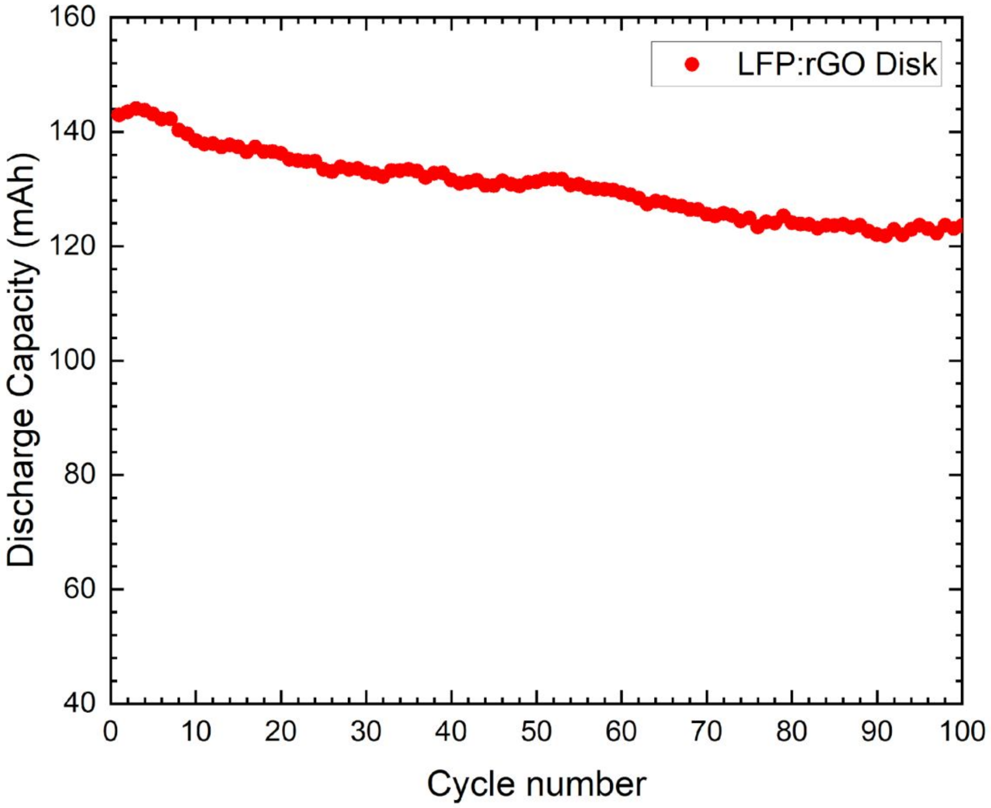

Figure S4. LFP:rGO disk specimen discharge tracked over 100 cycles.

References

(1) Nosé, S. A unified formulation of the constant temperature molecular dynamics methods. *The Journal of chemical physics* **1984**, *81* (1), 511-519.
(2) Hoover, W. G. Canonical dynamics: Equilibrium phase-space distributions. *Physical review A* **1985**, *31* (3), 1695.
(3) Monkhorst, H. J.; Pack, J. D. Special points for Brillouin-zone integrations. *Physical review B* **1976**, *13* (12), 5188.
(4) Perdew, J. P.; Burke, K.; Ernzerhof, M. Generalized gradient approximation made simple. *Physical review letters* **1996**, *77* (18), 3865.
(5) Kresse, G.; Joubert, D. From ultrasoft pseudopotentials to the projector augmented-wave method. *Physical review b* **1999**, *59* (3), 1758.
